# Discovery of three rare acremonium-like fungi in the rhizosphere of Gaultheria leucocarpa var. yunnanensis resolves the sister relationship between *Paraneoaraneomyces* and *Subuliphorum* (Clavicipitaceae, Hypocreales)

**DOI:** 10.3897/mycokeys.133.192083

**Published:** 2026-06-05

**Authors:** Yao Wang, Zhi-Qin Wang, Hui Chen, Li-Ping Zhu, Fan Yang, Shao-Huan Liu

**Affiliations:** 1 State Key Laboratory of Discovery and Utilization of Functional Components in Traditional Chinese Medicine & School of Pharmaceutical Sciences, Guizhou Medical University, Guian New District, Guiyang, Guizhou 561113, China State Key Laboratory of Discovery and Utilization of Functional Components in Traditional Chinese Medicine & School of Pharmaceutical Sciences, Guizhou Medical University Guiyang China; 2 The High Efficacy Application of Natural Medicinal Resources Engineering Center of Guizhou Province, Guizhou Medical University, Guian New District, Guiyang, Guizhou 561113, China The High Efficacy Application of Natural Medicinal Resources Engineering Center of Guizhou Province, Guizhou Medical University Guiyang China; 3 College of Biological Science and Food Engineering, Southwest Forestry University, Kunming, 650224, China College of Biological Science and Food Engineering, Southwest Forestry University Kunming China

**Keywords:** Clavicipitaceae, fungal taxonomy, multi-locus phylogeny, new species, rhizosphere fungi

## Abstract

Rhizosphere soils represent an underexplored reservoir of fungal diversity. In this study, three rare acremonium-like fungi were isolated from the rhizosphere of Gaultheria leucocarpa var. yunnanensis in Guizhou, China. Using a polyphasic taxonomic approach that integrates morphological observations with six-locus phylogenetic analyses (ITS, SSU, LSU, *tef*-*1α*, *rpb1*, and *rpb2*), the isolates were determined to represent two novel species and one new habitat record. *Paraneoaraneomyces guizhouensis* and *Subuliphorum cylindrosporum* are proposed as new species, while the rare species *Subuliphorum camptosporum* is reported from the plant rhizosphere for the first time. Phylogenetic analyses provide strong support for resolving *Paraneoaraneomyces* and *Subuliphorum* as distinct sister genera, a relationship that was previously unresolved. Together with *Neoaraneomyces*, these genera form a well-defined lineage within Clavicipitaceae. This study expands the known diversity and ecological range of these rare genera, clarifies their phylogenetic placement within Clavicipitaceae, and highlights the importance of rhizosphere sampling in uncovering hidden fungal diversity.

## Introduction

*Acremonium* and morphologically similar hyphomycetes (commonly termed “acremonium-like” fungi) are ubiquitous and exhibit diverse ecological roles as saprobes, parasites, or endophytes across a wide range of substrates, including soil, plant tissues, insect hosts, and aquatic sediments (Hou et al. 2023). This group has attracted considerable attention due to its capacity to produce bioactive secondary metabolites with potential applications in industry, medicine, and biological control. However, their taxonomy within Ascomycota has long been problematic because of limited and morphologically plastic diagnostic characters, making a phylogenetic framework essential for accurate classification.

A comprehensive revision by Hou et al. (2023), based on 633 acremonium-like strains, significantly advanced the systematics of this group. Using a multi-locus phylogeny (ITS, LSU, *rpb2*, and *tef*-*1α*), Hou et al. (2023) established several new genera, including *Subuliphorum*, with *S. camptosporum* designated as the type species within Clavicipitaceae. This species, characterized by verticillately or basitonously branched conidiophores bearing long, slender phialides, has been reported from soil, insects, and nematodes, with the type strain originally isolated as an aerial contaminant (Gams 1971; Hou et al. 2023). Notably, *S. camptosporum* is a rarely reported lineage. Since its original description by Gams (1971), only five strains have been formally documented in the scientific literature, with isolates originating from Germany, Cuba, South Africa, and an unknown locality (Hou et al. 2023). Even when considering additional unpublished sequences deposited in public databases (e.g., NCBI GenBank) that likely belong to this species, the total number of known strains is still extremely limited, underscoring its global rarity in culture collections.

The family Clavicipitaceae (Hypocreales, Ascomycota) comprises more than 500 species in 52 genera, displaying remarkable ecological diversity, including saprotrophic, symbiotic, and pathogenic lifestyles associated with plants, fungi, soil, and invertebrates (Sung et al. 2007a; Kepler et al. 2012a; Mongkolsamrit et al. 2020, 2021; Gao et al. 2021; Chen et al. 2022). An additional genus *Paraneoaraneomyces* was subsequently described by Zhang et al. (2023), with *P. sinensis* as the type species, isolated from soil and phylogenetically sister to *Neoaraneomyces*. Morphologically, *Paraneoaraneomyces* is distinguished by solitary, straight to flexuous phialides arising from aerial hyphae and producing cymbiform to reniform conidia. While both *Subuliphorum* and *Paraneoaraneomyces* represent rare lineages within Clavicipitaceae (Hou et al. 2023; Zhang et al. 2023), their precise phylogenetic relationship and ecological breadth remain insufficiently resolved.

The rhizosphere soil, a hotspot for microbial diversity and plant–microbe interactions, represents a promising yet underexplored reservoir for novel fungal taxa. This study focuses on the rhizosphere of Gaultheria leucocarpa var. yunnanensis (Ericaceae), a medicinal plant known as “Tougu Xiang” in traditional Chinese medicine that has been extensively studied for its diverse bioactive compounds, including gaultherin, diterpenoids and lignans as well as its anti-inflammatory, antioxidant, and anti-rheumatoid arthritis activities (Zhang et al. 2006; Wang et al. 2021, 2022; Hu et al. 2022, 2024). However, the microbial communities associated with this plant, particularly rhizosphere fungi, remain largely unexplored. Investigating this niche may reveal novel taxa and provide insights into potential plant-microbial interactions.

In this study, we isolated several rare acremonium-like fungi from the rhizosphere of G. leucocarpa var. yunnanensis, representing three distinct taxa. Using an integrative taxonomic approach combining morphology and multi-locus phylogenetic analyses (ITS, SSU, LSU, *tef*-*1α*, *rpb1*, and *rpb2*), we aimed to: 1) describe *Paraneoaraneomyces guizhouensis* as a new species; 2) introduce *Subuliphorum cylindrosporum* as a new species and report *S. camptosporum* from a new habitat; and 3) clarify the phylogenetic relationship between *Subuliphorum* and *Paraneoaraneomyces*. Our results provide new insights into the diversity, ecology, and evolutionary relationships of acremonium-like fungi within Clavicipitaceae.

## Materials and methods

### Isolates

Fungal isolation was carried out from rhizosphere soil samples of G. leucocarpa var. yunnanensis collected in May 2025 in Guian New District, Guizhou Province, China. At each of the 15 sampling sites, five healthy plants with similar growth status were selected. Rhizosphere soil was collected from their roots located in the upper 10 cm of the soil profile. Specifically, root systems were vigorously shaken to dislodge loosely attached soil, and the remaining thin layer of soil firmly adhering within 2–3 mm of the root surface was retained as the rhizosphere fraction (Smalla et al. 2001). The rhizosphere soil from the five plants at each site was pooled to form one composite sample, yielding a total of 15 composite samples. Each composite sample was thoroughly homogenized and passed through a 2 mm sterile sieve to remove plant debris and stones. For fungal isolation, following a modified procedure of Wang et al. (2025), a 2 g subsample from each sieved composite sample was suspended in 20 mL of sterile water with glass beads and shaken at 200 rpm for 10 min. The suspension was diluted 100-fold, and 200 μL aliquots were spread onto three different solid media: onion garlic agar (OGA, prepared by boiling 20 g each of grated onion and garlic in 1 L distilled water for 1 h, filtering, and adding 2% agar), Czapek yeast extract agar (CYA), and potato dextrose agar (PDA). All media were supplemented with 50 mg/L rose bengal and 100 mg/L kanamycin to suppress bacterial and fast-growing fungal contaminants (Wang et al. 2025). Plates were incubated at 25 °C, and emerging filamentous fungal colonies were subcultured onto fresh PDA plates for purification. To obtain axenic cultures, monospore isolation was performed by spreading a spore suspension (adjusted to ~1 × 10^3^ spores/mL in sterile saline) on PDA and incubating at 25 °C. Purified strains were grown on PDA slants at 25 °C until full sporulation (Wang et al. 2023). For long-term preservation, dried culture specimens were prepared by air-drying mature colonies (grown on PDA at 25 °C for 14 days) over silica gel. These specimens were deposited as herbarium material in the Guizhou Medical University Herbarium (**GMB**). Living cultures are maintained in the Guizhou Medical University Culture Collection (**GMBC**).

### Morphological observations

Cultures were incubated on PDA at 25 °C for 14 days (GMBC 3011–3013) or 30 days (GMBC 3009–3010). Colony diameters were measured after 14 and 30 days. Macro-morphological features, including surface and reverse colors and textures, were recorded and photographed with a Canon 750 D digital camera (Canon Inc., Tokyo, Japan). For micro-morphology, mycelia grown on PDA were transferred to agar blocks (ca. 5 mm in diameter) on microscope slides, overlaid with a coverslip, and mounted in sterile water. Conidiophores, phialides, and conidia were examined and photographed using an Olympus BX53 compound microscope. At least 50 measurements per morphological structure were taken for each isolate.

### DNA extraction, amplification and sequencing

Genomic DNA was extracted using a commercial purification kit (Qiagen GmbH, Hilden, Germany) from axenic cultures grown on PDA for 14 days, according to the manufacturer’s instructions, and stored at –20 °C. Six genetic loci (ITS, SSU, LSU, *tef*-*1α*, *rpb1*, and *rpb2*) were amplified by polymerase chain reaction (PCR). The primer pairs used were: ITS5/ITS4 (White et al. 1990) for ITS; NS1/NS4 (White et al. 1990) and LR0R/LR5 (Vilgalys and Hester 1990; Hopple 1994) for SSU and LSU, respectively; 2218R/983F (Rehner and Buckley 2005) for *tef*-*1α*; RPB1-5'F/RPB1-5'R (Bischoff et al. 2006; Sung et al. 2007a) for *rpb1*; and fRPB2-5F/fRPB2-7cR (Liu et al. 1999) for *rpb2*. PCR amplification was performed in a final volume of 50 µL following the conditions described by Wang et al. (2023). Consensus sequences were generated by assembling and aligning forward and reverse sequencing reads using Geneious Prime 2022 (Biomatters Inc., New Zealand).

### Phylogenetic analyses

Newly generated sequences were deposited in GenBank (Table 1). Additional sequences of related taxa were retrieved from GenBank based on recent phylogenetic studies (e.g., Mongkolsamrit et al. 2020, 2021; Gao et al. 2021; Chen et al. 2022; Hou et al. 2023; Zhang et al. 2023; a full list is provided in Table 1). Individual alignments for the six loci (ITS, SSU, LSU, *tef*-*1α*, *rpb1*, and *rpb2*) were generated using the MAFFT v. 7 online server (https://mafft.cbrc.jp/alignment/server/; accessed on 10 February 2026) and manually refined where necessary. Group I introns observed in SSU sequences of some species were excluded from phylogenetic analyses, and gaps were treated as missing data. These alignments were then concatenated into a supermatrix (SSU + ITS + LSU + *tef*-*1α* + *rpb1* + *rpb2*) for phylogenetic inference. Maximum likelihood (ML) analysis was performed using IQ-TREE v.2.1.3 (Minh et al. 2020). The best-fit substitution model (TIM3+F+I+G4) was selected with ModelFinder (Kalyaanamoorthy et al. 2017), and branch support was evaluated with 5000 ultrafast bootstrap replicates (Hoang et al. 2017). For comparative purposes, an additional ML analysis was also conducted using RAxML v.8.2.12 under the GTRCAT model with 1000 standard bootstrap replicates (Stamatakis et al. 2008). Bayesian inference (BI) was conducted using MrBayes v. 3.2.6 (Ronquist et al. 2012). The best-fit substitution model (GTR+I+G) for each partition was determined with jModelTest v. 2.1.4 (Darriba et al. 2012). Two independent runs of four Markov chains were performed for 3 million generations, sampling every 1000^th^ generation. The first 25% of sampled trees were discarded as burn-in after confirming convergence (average standard deviation of split frequencies < 0.01), and posterior probabilities (PP) were calculated from the remaining trees. The resulting phylogeny was visualized and annotated using FigTree v.1.4.2 (http://tree.bio.ed.ac.uk/software/figtree; accessed on 10 February 2026).

**Table 1.** Species, voucher information, hosts/substrate, and corresponding GenBank accession numbers of the taxa used in this study.

Species	Voucher/Culture	Host/Substrate	Genbank accession number						Reference
			ITS	SSU	LSU	*tef-1α*	*rpb1*	*rpb2*	
*Aciculosporium oplismeni*	MAFF 246966^T^	*Oplismenus undulatifolius*	LC571760	N/A	LC571760	LC572040	N/A	LC572054	Tanaka et al. (2021)
*Aciculosporium take*	MAFF 241224	*Phyllostachys pubescens*	LC571753	N/A	LC571753	LC572034	N/A	LC572048	Tanaka et al. (2021)
*Aciculosporium take*	TNS-F-60465	*Phyllostachys pubescens*	LC571755	N/A	LC571756	LC572035	N/A	LC572049	Tanaka et al. (2021)
*Albacillium fuzhouense*	CGMCC3.27818^T^	Coleoptera (larva)	N/A	PQ425616	PQ425618	PQ469143	PQ469145	PQ469147	Lin et al. (2025)
*Albacillium fuzhouense*	CGMCC3.27815	Coleoptera (larva)	N/A	PQ425617	PQ425619	PQ469144	PQ469146	PQ469148	Lin et al. (2025)
*Albacillium hingganense*	CCTCC M 20232069^T^	Forest litters	OR740562	MN055707	OR740566	MN065771	OR769082	OR769081	Ding et al. (2024)
*Aschersonia confluens*	BCC 7961	N/A	JN049841	DQ372100	DQ384947	DQ384976	DQ384998	DQ452465	Kepler et al. (2012b)
*Aschersonia placenta*	BCC 7869	Hemiptera: scale insect	JN049842	EF469121	EF469074	EF469056	EF469085	EF469104	Sung et al. (2007a, 2007b); Kepler et al. (2012a)
*Atkinsonella hypoxylon*	B4728	*Danthonia spicata*	N/A	N/A	N/A	KP689546	N/A	KP689514	Christopher et al. (2014)
*Balansia epichloe*	A.E.G. 96-15a	Poaceae	JN049848	N/A	N/A	EF468743	EF468851	EF468908	Sung et al. (2007a); Kepler et al. (2012a)
*Balansia henningsiana*	A.E.G. 96-27a	*Panicum* sp.	JN049815	AY545723	AY545727	AY489610	AY489643	DQ522413	Spatafora et al. (2007); Kepler et al. (2012a)
*Claviceps fusiformis*	ATCC 26019	Poaceae	JN049817	DQ522539	U17402	DQ522320	DQ522366	N/A	Spatafora et al. (2007); Kepler et al. (2012a)
*Claviceps purpurea*	GAM 12885	Poaceae	U57669	N/A	AF543789	AF543778	N/A	DQ522417	Currie et al. (2003); Spatafora et al. (2007)
*Claviceps purpurea*	S.A. cp 11	Poaceae	N/A	EF469122	EF469075	EF469058	EF469087	EF469105	Sung et al. (2007a, 2007b)
*Collarina aurantiaca*	FMR 11134	Sediments of Ara River	KJ807178	N/A	KJ807181	N/A	N/A	N/A	Crous et al. (2014)
*Collarina aurantiaca*	FMR 11784	Sediments of Ara River	KJ807177	N/A	KJ807180	N/A	N/A	N/A	Crous et al. (2014)
*Conoideocrella luteorostrata*	NHJ 11343	Hemiptera: scale insect	JN049859	EF468995	EF468850	EF468801	EF468906	N/A	Sung et al. (2007a); Kepler et al. (2012a)
*Conoideocrella luteorostrata*	NHJ 12516	Hemiptera: scale insect	JN049860	EF468994	N/A	EF468800	EF468905	EF468946	Sung et al. (2007a); Kepler et al. (2012a)
*Conoideocrella tenuis*	NHJ 6293	Hemiptera: scale insect	JN049862	EU369112	EU369044	EU369029	EU369068	EU369087	Johnson et al. (2009); Kepler et al. (2012a)
*Corallocytostroma ornithocopreoides*	WAC 8705	*Astrebla pectinata*	N/A	N/A	N/A	LT216546	N/A	LT216620	Píchová et al. (2018)
*Dussiella tuberiformis*	J.F.White	*Arundinaria tecta*	N/A	N/A	N/A	JQ257027	JQ257015	JQ257020	Kepler et al. (2012b)
*Ephelis japonica*	CBS 236.64	N/A	MH858427	N/A	N/A	N/A	N/A	N/A	Vu et al. (2019)
*Ephelis japonica*	Eph.oryzae	*Oryza sativa*	AB038564	N/A	N/A	N/A	N/A	N/A	Tanaka et al. (2002)
*Ephelis tripsaci*	CBS 857.72^T^	Leaf sheath of *Tripsacum laxum*	KP859042	N/A	KP858978	N/A	N/A	N/A	Hernández-Restrepo et al. (2016)
*Epichloë elymi*	C.Schardl 760	N/A	N/A	N/A	AY986924	AY986951	DQ000352	N/A	Chaverri et al. (2005)
*Epichloë typhina*	ATCC 56429	*Fragaria* sp. (Rosaceae)	JN049832	N/A	U17396	AF543777	AY489653	DQ522440	Rehner and Samuels (1995); Currie et al. (2003); Spatafora et al. (2007); Kepler et al. (2012b)
*Heteroepichloe bambusae*	Ba-01	*Gigantochloa* sp.	AB065426	N/A	N/A	N/A	N/A	N/A	Tanaka et al. (2002)
*Heteroepichloe bambusae*	Bo-01	*Gigantochloa* sp.	AB065428	N/A	N/A	N/A	N/A	N/A	Tanaka et al. (2002)
*Heteroepichloe sasae*	E.sasae-H	*Sasa* sp.	AB065432	N/A	N/A	N/A	N/A	N/A	Tanaka et al. (2002)
*Heteroepichloe sasae*	E.sasae-N	*Sasa* sp.	AB065431	N/A	N/A	N/A	N/A	N/A	Tanaka et al. (2002)
*Keithomyces carneus*	CBS 239.32^T^	Sand dune	NR_131993	EF468988	NG_057769	EF468789	EF468894	EF468938	Sung et al. (2007a)
*Keithomyces* sp.	CBS 126563	Soil	MT078883	MT078871	MT078856	N/A	MT078864	MT078921	Mongkolsamrit et al. (2020)
*Marquandomyces marquandii*	CBS 182.27	Soil	MH854923	EF468990	MH866418	EF468793	EF468899	EF468942	Sung et al. (2007a); Vu et al. (2019)
*Marquandomyces* sp.	CBS 127132	Soil	MT078882	MT078872	MT078857	N/A	MT078865	MT078922	Mongkolsamrit et al. (2020)
*Metapochonia bulbillosa*	JCM 18596	Soil	AB709836	AB758252	AB709809	AB758460	AB758663	AB758690	Nonaka et al. (2013)
*Metapochonia bulbillosa*	CBS 145.70^T^	Root of *Picea abies*	MH859529	AF339591	AF339542	EF468796	EF468902	EF468943	Sung et al. (2001); Sung et al. (2007a); Vu et al. (2019)
*Metapochonia cordycipiticonsociata*	CGMCC 3.17365	*Hepialus armoricanus*	KM263569	KM263572	KM263573	KM263584	KM263576	KM263579	Huang et al. (2015)
*Metapochonia cordycipiticonsociata*	CGMCC 3.17366	*Hepialus armoricanus*	KM263567	KM263570	KM263574	KM263582	KM263578	KM263580	Huang et al. (2015)
*Metapochonia goniodes*	CBS 891.72^T^	Nematode	AJ292409	AF339599	AF339550	DQ522354	DQ522401	DQ522458	Zare et al. (2000); Sung et al. (2001); Spatafora et al. (2007)
*Metapochonia microbactrospora*	CBS 101433^T^	N/A	AJ292408	N/A	AF339538	KJ398794	N/A	KJ398701	Zare et al. (2000); Kepler et al. (2014)
*Metapochonia rubescens*	CBS 464.88^T^	*Heterodera avenae* (Nematoda)	MH862138	AF339615	MH873830	EF468797	EF468903	EF468944	Sung et al. (2001); Sung et al. (2007a); Vu et al. (2019)
*Metapochonia rubescens*	JCM 18620	Soil	AB709859	AB758247	AB709832	AB758455	AB758658	AB758685	Nonaka et al. (2013)
Metapochonia suchlasporia var. catenata	CBS 248.83	Nematode eggs	MH861579	N/A	MH873310	KJ398789	N/A	KJ398696	Kepler et al. (2014); Vu et al. (2019)
Metapochonia suchlasporia var. catenata	CBS 251.83	Nematode eggs	MH861580	N/A	MH873311	KJ398790	N/A	KJ398697	Kepler et al. (2014); Vu et al. (2019)
*Metarhiziopsis microspora*	CEHS133a	*Fiorinia externa*	EF464589	N/A	EF464571	N/A	N/A	N/A	Marcelino et al. (2009)
*Metarhiziopsis microspora*	INEHS133a	*Fiorinia externa*	EF464583	N/A	EF464572	N/A	N/A	N/A	Marcelino et al. (2009)
*Metarhizium anisopliae*	CBS 130.71^T^	*Avena sativa*	MT078884	MT078868	MT078853	MT078845	MT078861	MT078918	Mongkolsamrit et al. (2020)
*Metarhizium flavoviride*	CBS 125.65	Soil	MT078885	MT078869	MT078854	MT078846	MT078862	MT078919	Mongkolsamrit et al. (2020)
*Metarhizium flavoviride*	CBS 218.56^T^	Coleoptera	MH857590	N/A	MH869139	KJ398787	KJ398598	KJ398694	Kepler et al. (2014); Vu et al. (2019)
*Moelleriella phyllogena*	CUP 067785	Insect	N/A	N/A	EU392610	EU392674	EU392726	N/A	Chaverri et al. (2008)
*Moelleriella phyllogena*	CUP 067793	Insect	N/A	N/A	EU392608	EU392672	EU392724	N/A	Chaverri et al. (2008)
*Moelleriella umbospora*	CUP 067817^T^	N/A	N/A	N/A	EU392628	EU392688	EU392740	N/A	Chaverri et al. (2008)
*Morakotia fusca*	BCC 64125	Plant	N/A	N/A	KY794862	KY794857	N/A	N/A	Mongkolsamrit et al. (2021)
*Morakotia fusca*	BCC 79272^T^	Plant	N/A	N/A	KY794861	KY794856	KY794865	N/A	Mongkolsamrit et al. (2021)
*Mycophilomyces periconiae*	CPC 27558	N/A	KY173418	N/A	KY173509	N/A	N/A	N/A	Crous et al. (2014)
*Myriogenospora atramentosa*	A.E.G 96-32	Plant	N/A	AY489701	AY489733	AY489628	AY489665	DQ522455	Castlebury et al. (2004); Spatafora et al. (2007)
*Neoaraneomyces araneicola*	DY101711^T^	Spider	MW730520	N/A	MW730609	MW753033	MW753024	MW753026	Chen et al. (2022)
*Neoaraneomyces araneicola*	DY101712	Spider	MW730522	N/A	MW730610	MW753034	N/A	MW753027	Chen et al. (2022)
*Neobarya parasitica*	Marson s/n	Bertia	KP899626	N/A	N/A	N/A	N/A	N/A	Lawrey et al. (2015)
*Niesslia exilis*	CBS 560.74	On decaying needle of *Pinus sylvestris*	MG827005	AY489688	AY489720	AY489614	AY489647	N/A	Castlebury et al. (2004)
*Nigelia aurantiaca*	BCC 13019	Lepidoptera (larva)	N/A	GU979939	GU979948	GU979957	GU979966	GU979971	Luangsa-ard et al. (2017)
*Nigelia martialis*	EFCC 6863	Lepidoptera	N/A	N/A	JF415974	JF416016	N/A	N/A	Kepler et al. (2012a)
*Orbiocrella petchii*	NHJ 6209	Hemiptera: scale insect	JN049861	EU369104	EU369039	EU369023	EU369061	EU369081	Johnson et al. (2009); Kepler et al. (2012a)
*Orbiocrella petchii*	NHJ 6240	Hemiptera: scale insect	N/A	EU369103	EU369038	EU369022	EU369060	EU369082	Johnson et al. (2009)
*Papiliomyces sinensis*	GMBC 3053^T^	*Napialus* larva	PQ636503	PQ636500	PQ636506	PQ660655	PQ660658	PQ660661	Chen et al. (2025)
*Papiliomyces sinensis*	GMBC 3054	*Napialus* larva	PQ636504	PQ636501	PQ636507	PQ660656	PQ660659	PQ660662	Chen et al. (2025)
*Papiliomyces shibinensis*	GZUH SB13050311^T^	Lepidoptera larva	KR153585	KR153588	N/A	KR153589	N/A	N/A	Wen et al. (2015)
*Parametarhizium changbaiense*	CGMCC 19143^T^	Litters of forest	MN589741	MN590231	MN589994	MN908589	MN917168	MT921829	Gao et al. (2021)
*Parametarhizium hingganense*	CGMCC 19144^T^	Litters of forest	MN055703	MN055706	MN061635	MN065770	MN917170	MT939494	Gao et al. (2021)
***Paraneoaraneomyces guizhouensis***	**GMBC 3009^T^**	**Soil**	**PV701288**	**PV701283**	**PV701293**	**PV837501**	**PV837506**	**PV837511**	**This study**
***Paraneoaraneomyces guizhouensis***	**GMBC 3010**	**Soil**	**PV701289**	**PV701284**	**PV701294**	**PV837502**	**PV837507**	**PV837512**	**This study**
*Paraneoaraneomyces sinensis*	ZY 22.006^T^	Soil	OQ709254	OQ709248	OQ709260	OQ719626	N/A	OQ719621	Zhang et al. (2023)
*Paraneoaraneomyces sinensis*	ZY 22.007	Soil	OQ709255	OQ709249	OQ709261	OQ719627	N/A	OQ719622	Zhang et al. (2023)
*Paraneoaraneomyces sinensis*	ZY 22.008	Soil	OQ709256	OQ709250	OQ709262	OQ719628	N/A	OQ719623	Zhang et al. (2023)
*Parepichloe cinerea*	Ne-01	*Sporobolus* sp.	AB065425	N/A	N/A	N/A	N/A	N/A	Tanaka et al. (2002)
*Pleurocordyceps aurantiaca*	MFLUCC 17-2113^T^	*Ophiocordyceps barnesii*	MG136916	MG136904	MG136910	MG136875	MG136866	MG136870	Xiao et al. (2018)
*Pleurocordyceps marginaliradians*	MFLU 17-1582^T^	Lepidoptera: Cossidae	MG136920	MG136908	MG136914	MG136878	MG136869	MG271931	Wang et al. (2021)
*Periglandula ipomoeae*	IasaF13	*Ipomoea asarifolia*	N/A	N/A	N/A	KP689568	KC113320	KP689517	Christopher et al. (2014)
*Pochonia boninensis*	JCM 18597	Soil	AB709858	AB758255	AB709831	AB758463	AB758666	AB758693	Nonaka et al. (2013)
*Pochonia chlamydosporia*	CBS 101244	Mollusca	JN049821	DQ522544	DQ518758	DQ522327	DQ522372	DQ522424	Spatafora et al. (2007); Kepler et al. (2012b)
Pochonia chlamydosporia var. catenulata	CBS 504.66^T^	Soil	AJ292398	AF339593	AF339544	EF469069	EF469098	EF469120	Zare et al. (2000); Sung et al. (2001); Sung et al. (2007a)
Pochonia chlamydosporia var. catenulata	JCM 18598	Soil	AB709837	AB758248	AB709810	AB758456	AB758659	AB758686	Nonaka et al. (2013)
Pochonia chlamydosporia var. catenulata	JCM 18600	Soil	AB709839	AB758266	AB709812	AB758474	AB758677	AB758704	Nonaka et al. (2013)
Pochonia chlamydosporia var. chlamydosporia	JCM 18605	Soil	AB709844	AB758261	AB709817	AB758469	AB758672	AB758699	Nonaka et al. (2013)
Pochonia chlamydosporia var. chlamydosporia	JCM 18607	Soil	AB709846	AB758270	AB709819	AB758478	AB758681	AB758708	Nonaka et al. (2013)
Pochonia chlamydosporia var. ellipsospora	JCM 18609^T^	Soil	AB709848	AB758257	AB709821	AB758465	AB758668	AB758695	Nonaka et al. (2013)
Pochonia chlamydosporia var. ellipsospora	JCM 18611	Soil	AB709850	AB758265	AB709823	AB758473	AB758676	AB758703	Nonaka et al. (2013)
Pochonia chlamydosporia var. spinulospora	JCM 18613^T^	Soil	AB709854	AB758258	AB709827	AB758466	AB758669	AB758696	Nonaka et al. (2013)
Pochonia chlamydosporia var. spinulospora	JCM 18619	Soil	AB709857	AB758272	AB709830	AB758480	AB758683	AB758710	Nonaka et al. (2013)
*Pochonia sinensis*	ZY 22.009^T^	Soil	OQ709257	OQ709251	OQ709263	OQ719629	N/A	OQ719624	Zhang et al. (2023)
*Pochonia sinensis*	ZY 22.010	Soil	OQ709258	OQ709252	OQ709264	OQ719630	N/A	OQ719625	Zhang et al. (2023)
*Purpureomyces khaoyaiensis*	BCC 1376^T^	Lepidoptera	KX983460	KX983468	KX983462	KX983457	N/A	KX983465	Luangsa-ard et al. (2017)
*Purpureomyces maesotensis*	BCC 89300^T^	Lepidoptera larva	MN781917	N/A	MN781876	MN781733	MN781778	N/A	Mongkolsamrit et al. (2020)
*Purpureomyces maesotensis*	BCC 88441	Lepidoptera larva	MN781916	N/A	MN781877	MN781734	MN781779	MN781824	Mongkolsamrit et al. (2020)
*Regiocrella camerunensis*	ARSEF 7682^T^	Scale insects	N/A	N/A	DQ118735	DQ118743	DQ127234	N/A	Chaverri et al. (2005)
*Rotiferophthora angustispora*	CBS 101437	Rotifer (Rotifera)	AJ292412	AF339584	AF339535	AF543776	DQ522402	DQ522460	Zare et al. (2000); Sung et al. (2001); Currie et al. (2003); Spatafora et al. (2007)
*Samuelsia chalalensis*	CUP 067856^T^	N/A	N/A	N/A	EU392637	EU392691	EU392743	N/A	Chaverri et al. (2008)
*Samuelsia mundiveteris*	BCC 40021	Hemiptera: Scale insect nymphs	N/A	N/A	GU552152	GU552145	N/A	N/A	Mongkolsamrit et al. (2011)
*Samuelsia rufobrunnea*	CUP 067858^T^	N/A	N/A	N/A	AY986918	AY986944	DQ000345	N/A	Chaverri et al. (2005)
*Shimizuomyces paradoxus*	EFCC 6279	*Smilax sieboldii*	JN049847	EF469131	EF469084	EF469071	EF469100	EF469117	Sung et al. (2007a, 2007b); Kepler et al. (2012b)
*Shimizuomyces paradoxus*	EFCC 6564	*Smilax sieboldii*	N/A	EF469130	EF469083	EF469072	EF469101	EF469118	Sung et al. (2007a, 2007b)
*Subuliphorum camptosporum*	CBS 677.74	Soil	OQ429876	N/A	OQ430127	OQ471208	N/A	OQ454276	Hou et al. (2023)
*Subuliphorum camptosporum*	CBS 756.69^T^	Aerial contaminant	OQ429878	N/A	OQ430129	OQ471210	N/A	OQ454278	Hou et al. (2023)
*Subuliphorum camptosporum*	CBS 835.91	Insect	OQ429877	N/A	OQ430128	OQ471209	N/A	OQ454277	Hou et al. (2023)
*Subuliphorum camptosporum*	CBS 890.85	Soil	OQ429875	N/A	OQ430126	OQ471207	N/A	OQ454275	Hou et al. (2023)
***Subuliphorum camptosporum***	**GMBC 3011**	**Soil**	**PV701290**	**PV701285**	**PV701295**	**PV837503**	**PV837508**	**PV837513**	**This study**
***Subuliphorum cylindrosporum***	**GMBC 3012^T^**	**Soil**	**PV701291**	**PV701286**	**PV701296**	**PV837504**	**PV837509**	**PV837514**	**This study**
***Subuliphorum cylindrosporum***	**GMBC 3013**	**Soil**	**PV701292**	**PV701287**	**PV701297**	**PV837505**	**PV837510**	**PV837515**	**This study**
*Sungia yongmunensis*	EFCC 2131^T^	Lepidopteran pupa	JN049856	EF468977	EF468833	EF468770	EF468876	N/A	Sung et al. (2007a); Kepler et al. (2012a)
*Sungia yongmunensis*	EFCC 2135	Lepidopteran pupa	N/A	EF468979	EF468834	EF468769	EF468877	N/A	Sung et al. (2007a)
*Tyrannicordyceps fratricida*	TNS 19011	Fungi	N/A	JQ257022	JQ257023	JQ257028	JQ257016	JQ257021	Kepler et al. (2012b)
*Ustilaginoidea dichromenae*	MRL IB9228	Plant	N/A	N/A	N/A	JQ257025	JQ257013	JQ257018	Kepler et al. (2012b)
*Ustilaginoidea virens*	ATCC 16180	Plant	N/A	N/A	N/A	JQ257026	JQ257014	JQ257019	Kepler et al. (2012b)
*Ustilaginoidea virens*	MAFF 240421	Plant	JQ349068	N/A	JQ257011	JQ257024	N/A	JQ257017	Kepler et al. (2012b)
*Yosiokobayasia kusanagiensis*	TNS-F18494	Coleoptera	JN049873	JF415954	JF415972	JF416014	JN049890	N/A	Kepler et al. (2012a)

Boldface: data generated in this study; ^T^: ex-type culture.

To further investigate the phylogenetic relationships among the genera *Paraneoaraneomyces*, *Subuliphorum*, and *Neoaraneomyces*, a separate analysis was conducted focusing on all available species within these three genera. The same six loci (ITS, SSU, LSU, *tef*-*1α*, *rpb1*, and *rpb2*) were employed. *Metapochonia bulbillosa* (CBS 145.70) and *Metapochonia goniodes* (CBS 891.72) were selected as outgroup taxa based on their phylogenetic position in previous studies (e.g., Zhang et al. 2023). Alignments for each locus were generated and concatenated as described above. Maximum likelihood (ML) analyses were performed using both IQ-TREE v.2.1.3 and RAxML v.8.2.12 under the same settings as the overall analysis, with bootstrap support evaluated via 5000 ultrafast replicates (IQ-TREE) and 1000 standard replicates (RAxML). Bayesian inference (BI) was conducted with MrBayes v.3.2.6 using parameters identical to those described for the combined dataset. Additionally, single-locus genealogies for each of the six markers were constructed under the ML criterion using RAxML v.8.2.12 to assess potential conflicts among individual gene trees.

## Results

### Phylogenetic analyses

**Figure 1.**
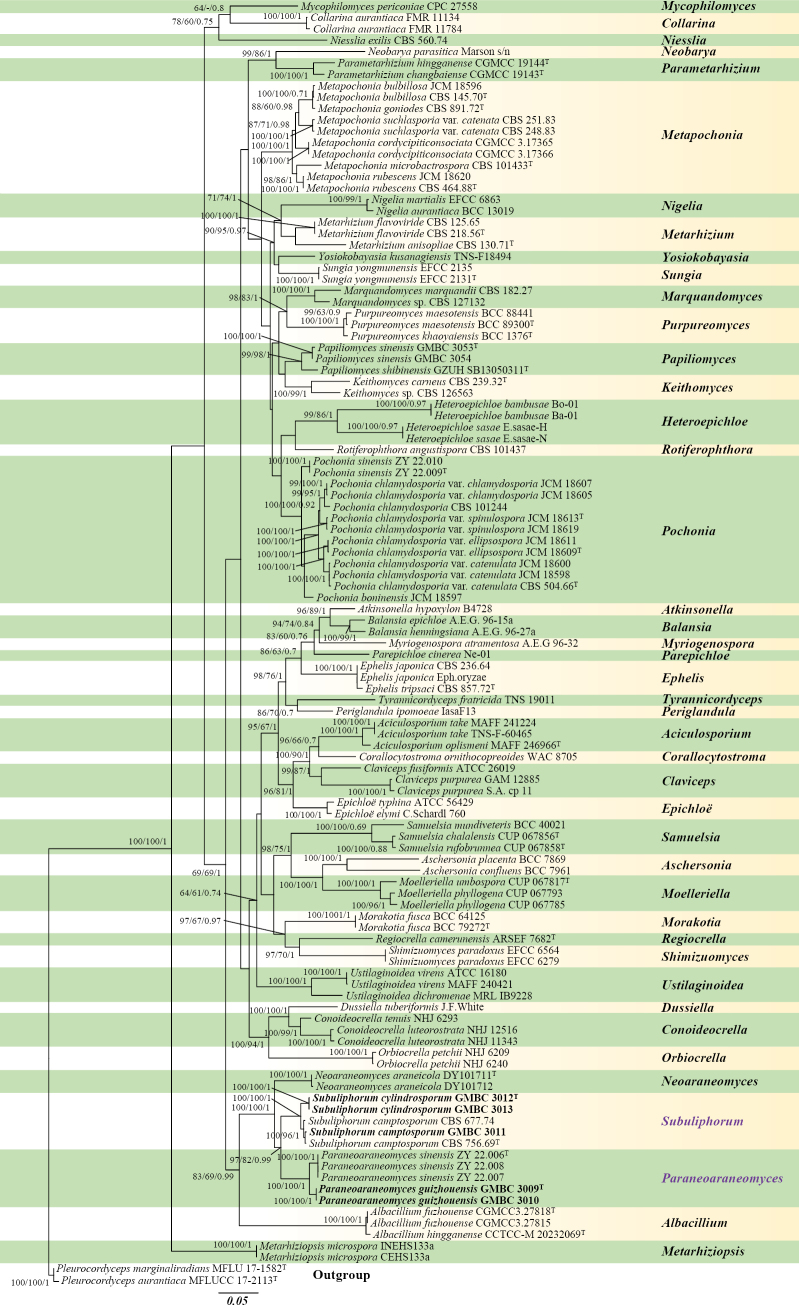
Phylogenetic relationships within Clavicipitaceae inferred from a concatenated alignment of six loci (SSU + ITS + LSU + *tef*-*1α* + *rpb1* + *rpb2*) under the maximum likelihood criterion. Nodal support is indicated as follows: IQ-TREE ultrafast bootstrap (BS_IQ_) > 60% / RAxML standard bootstrap (BS_RAx_) > 60%, and Bayesian posterior probability (PP) > 0.70. Strains examined in this study are highlighted in bold.

To resolve the phylogenetic positions of the obtained isolates, analyses were based on a concatenated dataset of six loci (SSU + ITS + LSU + *tef*-*1α* + *rpb1* + *rpb2*). The final alignment comprised 6,227 characters (including gaps), with the length of individual partitions as follows: SSU, 1,675 bp; ITS, 859 bp; LSU, 934 bp; *tef*-*1α*, 948 bp; *rpb1*, 797 bp; and *rpb2*, 1,014 bp. *Pleurocordyceps aurantiaca* (MFLUCC 17-2113) and *P. marginaliradians* (MFLU 17-1582) were designated as outgroup taxa. Both maximum-likelihood (ML) and Bayesian inference (BI) analyses yielded highly congruent topologies, recovering 44 clades representing major lineages within Clavicipitaceae, with *Pleurocordyceps* species (Polycephalomycetaceae) positioned as outgroup (Fig. 1).

Within this broad phylogenetic framework, our isolates were precisely placed. Strains GMBC 3009 and GMBC 3010 formed a distinct, well-supported lineage (BS_IQ_/BS_RAx_/PP = 100%/100%/1) sister to *P. sinensis*, confirming their status as the novel species *P. guizhouensis*. Within *Subuliphorum*, *S. cylindrosporum* sp. nov. (GMBC 3012, GMBC 3013) was resolved as sister to *S. camptosporum* with strong statistical support (100%/100%/1). Strain GMBC 3011 also was robustly identified as *S. camptosporum*, clustering with its ex-type strain CBS 756.69 and strain CBS 677.74 (100%/96%/1), which constitutes a new habitat record for this rare species. In the resulting phylogeny, all *Paraneoaraneomyces* species formed a well-supported clade (100%/100%/1), and all *Subuliphorum* species formed another well-supported clade (100%/100%/1). These two genera were resolved as sisters—with strong support in the IQ-TREE analysis (97%) and Bayesian inference (0.99), and moderate support in the RAxML analysis (82%)—and together with *Neoaraneomyces* constituted a distinct, fully supported lineage within Clavicipitaceae (100%/100%/1) (Fig. 1).

**Figure 2.**
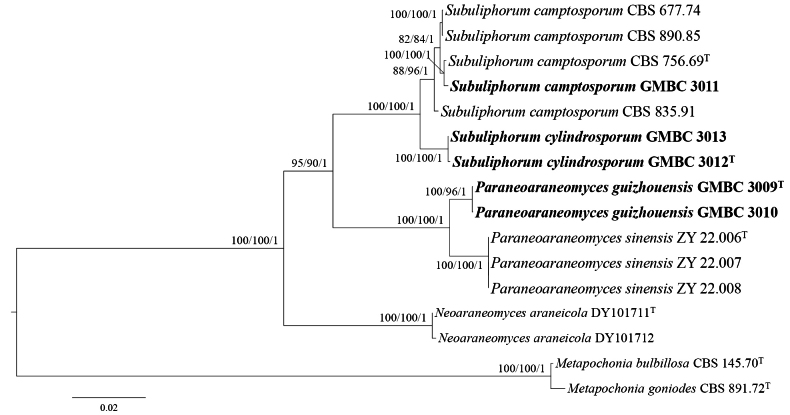
Phylogenetic relationships among *Paraneoaraneomyces*, *Subuliphorum*, and *Neoaraneomyces* inferred from a concatenated six-locus alignment (SSU + ITS + LSU + *tef*-*1α* + *rpb1* + *rpb2*) using maximum likelihood. Support values (BS_IQ_/BS_RAx_/PP) are shown at nodes. Strains examined in this study are highlighted in bold.

To further resolve the interspecific relationships and confirm the monophyly of the newly proposed species within the *Paraneoaraneomyces*–*Subuliphorum*–*Neoaraneomyces* clade, a separate phylogenetic analysis was conducted focusing exclusively on all available taxa from these three genera. The same six-locus dataset was employed, with *Metapochonia bulbillosa* (CBS 145.70) and *M. goniodes* (CBS 891.72) as outgroup taxa (Fig. 2). This focused analysis, based on the same six-locus dataset but with a different outgroup selection, yielded congruent topologies, although nodal support values showed minor variations: *Paraneoaraneomyces* and *Subuliphorum* formed a sister clade (95%/90%/1; Fig. 2), consistent with their relationship inferred in the broader analysis (97%/82%/0.99; Fig. 1). Together with *Neoaraneomyces*, they constituted a fully supported lineage (100%/100%/1).

Single-locus genealogies for each of the six markers were constructed under the ML criterion to assess potential conflicts among individual genes (Suppl. material 1: figs S1–S6). Although some topological inconsistencies were observed, the novel species were consistently recovered as distinct clades. For the protein-coding genes (*tef*-*1α*, *rpb1*, *rpb2*), the topologies were largely congruent with the concatenated tree: *Paraneoaraneomyces* and *Subuliphorum* clustered together, forming a lineage with *Neoaraneomyces* (Suppl. material 1: figs S4–S6). In contrast, the ITS and LSU trees placed *Subuliphorum* closer to *Neoaraneomyces*, with *Paraneoaraneomyces* as sister to this clade (Suppl. material 1: figs S2, S3). The SSU gene tree, although incomplete due to missing sequence data for *Neoaraneomyces*, also supported a close relationship between *Paraneoaraneomyces* and *Subuliphorum* (Suppl. material 1: fig. S1). Despite these discrepancies, both novel species received consistent multi-locus support. *P. guizhouensis* was sister to *P. sinensis* in the ITS, *tef*-*1α*, and *rpb2* trees (Suppl. material 1: figs S2, S4, S6). *S. cylindrosporum* formed a robust sister clade to *S. camptosporum* in the ITS, LSU, *tef*-*1α*, *rpb1*, and *rpb2* trees (Suppl. material 1: figs S2–S6). These results demonstrate that while single-gene trees (particularly ITS and LSU) may yield conflicting topologies, multi-locus analyses provide a robust framework for resolving relationships among closely related genera in Clavicipitaceae. The consistent placement of the two novel species across multiple markers further validates their taxonomic status. Moreover, the observed gene-tree conflicts underscore the importance of incorporating protein-coding loci in phylogenetic reconstructions of this group.

### Taxonomy

Five strains isolated from the rhizosphere soil of Gaultheria leucocarpa var. yunnanensis in Guizhou Province, China (Guian New District), were determined to represent three species. Two of these are proposed as new species: *Paraneoaraneomyces guizhouensis* (strains GMBC 3009 and GMBC 3010) and *Subuliphorum cylindrosporum* (strains GMBC 3012 and GMBC 3013). The fifth strain, GMBC 3011, was identified as *Subuliphorum camptosporum*. A detailed redescription is omitted here, and key taxonomic and ecological notes are provided below.

#### Paraneoaraneomyces guizhouensis

Taxon classificationFungiHypocrealesClavicipitaceae

Y. Wang & H. Chen sp. nov.

981D2B6B-0202-5318-B305-D0C596F1B210

862359

Fig. 3

##### Etymology.

The specific epithet refers to Guizhou Province, China, where the holotype was collected.

**Figure 3.**
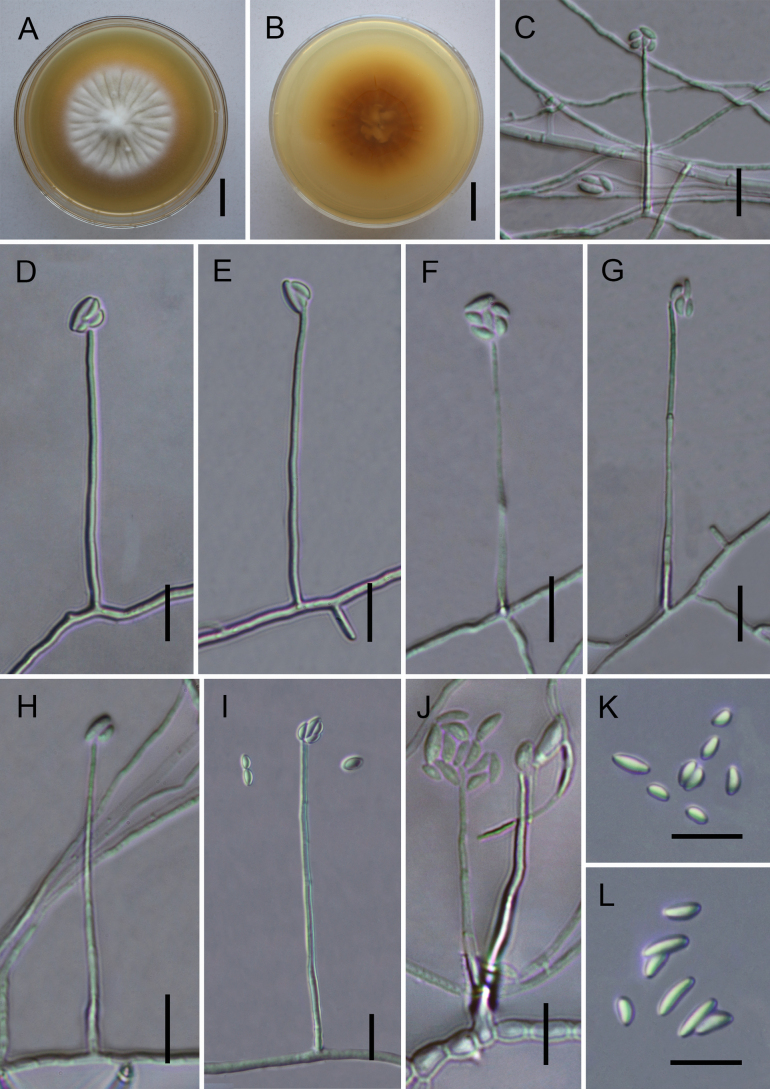
Morphology of *Paraneoaraneomyces guizhouensis*. **A, B**. Colonies on PDA (front and reverse views); **C–J**. Hyphae bearing phialides and conidia; **K, L**. Detached conidia. Scale bars: 2 cm (**A, B**); 10 μm (**C–L**).

##### Type.

China, • Guizhou Province, Guian New District (26°23'48"N, 106°27'08"E, alt. 1235 m), isolated from the rhizosphere soil of Gaultheria leucocarpa var. yunnanensis, May 2025, Yao Wang (holotype a dried culture GMB 3009); ex-type living culture GMBC 3009.

##### Description.

***Colonies*** on PDA reaching 35–40 mm in diameter in 30 days at 25 °C, white, slightly raised at center, plicate, nearly circular with regular margin; reverse pale yellow. ***Hyphae*** hyaline, smooth, branched, septate, 1.0–2.2 μm wide. ***Phialides*** arising from aerial or vegetative hyphae, solitary or occasionally in whorls, straight to slightly curved, tapering toward the apex, base slightly enlarged, smooth, hyaline, 28.0–69.0 × 0.8–1.9 μm (x̄ = 56.0 × 1.3, n = 50). ***Conidia*** formed at phialide apices, often aggregated in small globose heads, cymbiform to reniform, smooth-walled, aseptate, 3.5–7.0 × 1.5–2.5 μm (x̄ = 4.9 × 2.0, n = 50). ***Sexual morph*** not observed.

##### Other material examined.

China, • Guizhou Province, Guian New District (same locality and habitat as the holotype), May 2025, Yao Wang (living culture GMBC 3010).

##### Substrate.

Rhizosphere soil.

##### Distribution.

Known only from Guizhou Province, China.

##### Notes.

In the six-locus phylogeny (ITS, SSU, LSU, *tef*-*1α*, *rpb1*, and *rpb2*), strains GMBC 3009 (ex-type) and GMBC 3010 of *Paraneoaraneomyces guizhouensis* formed a distinct clade sister to *P. sinensis*, with strong statistical support (100%/100%/1; Figs 1, 2). Jeewon and Hyde (2016) suggested that nucleotide differences exceeding 1.5% in ITS and protein-coding genes may indicate species-level divergence. BLASTn comparisons of sequences from the ex-type strain with those of *P. sinensis* were conducted for ITS, *tef*-*1α*, and *rpb2* (as *rpb1* data for *P. sinensis* are unavailable). The results revealed 3/531 bp (0.56%) divergence in ITS, below the suggested threshold; 17/937 bp (1.81%) in *tef*-*1α*; and 42/1004 bp (4.18%) in *rpb2*, with the latter two exceeding the recommended cut-off. These molecular differences are supported by clear morphological discontinuities. *Paraneoaraneomyces guizhouensis* produces longer phialides (28.0–69.0 × 0.8–1.9 μm, x̄ = 56.0 × 1.3 μm vs. 19.0–34.0 × 0.5–1.5 μm, x̄ = 27.0 × 1.1 μm in *P. sinensis*) and larger conidia (3.5–7.0 × 1.5–2.5 μm, x̄ = 4.9 × 2.0 μm vs. 3.0–5.5 × 1.0–1.5 μm, x̄ = 4.3 × 1.4 μm; Zhang et al. 2023). Additionally, phialides in the new species occasionally arise in whorls (Fig. 3J), a feature not observed in *P. sinensis*. The combination of phylogenetic evidence, multi-locus genetic divergence (particularly in protein-coding genes), and consistent morphological differences supports the recognition of *P. guizhouensis* as a distinct species.

#### Subuliphorum cylindrosporum

Taxon classificationFungiHypocrealesClavicipitaceae

Y. Wang & H. Chen sp. nov.

2A4AB774-F1A2-5C8E-B16A-56E6839068F3

862360

Fig. 4

##### Etymology.

The name refers to the cylindrical conidia, a distinctive morphological feature of this species.

**Figure 4.**
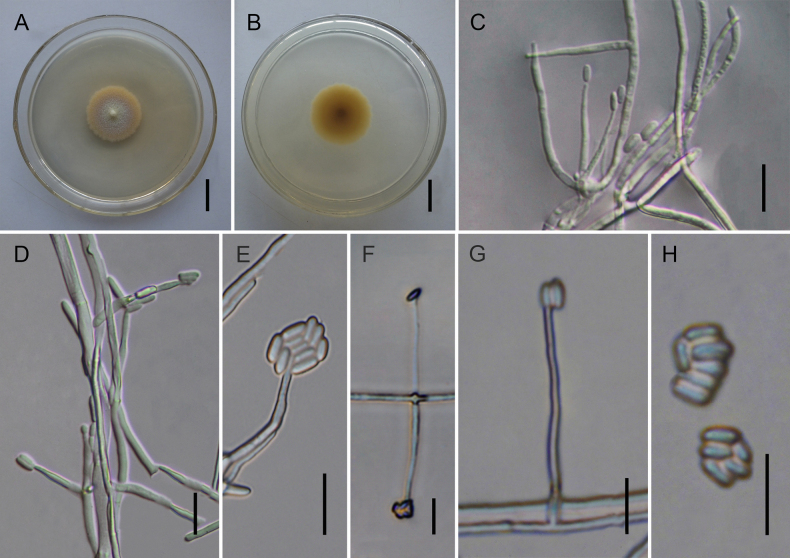
Morphology of *Subuliphorum cylindrosporum*. **A, B**. Colonies on PDA (front and reverse views); **C–G**. Conidiophores, phialides, and conidia; **H**. Detached conidia. Scale bars: 2 cm (**A, B**); 10 μm (**C–H**).

##### Type.

China, • Guizhou Province, Guian New District (26°23'52"N, 106°27'10"E, alt. 1230 m), isolated from the rhizosphere soil of Gaultheria leucocarpa var. yunnanensis, May 2025, Yao Wang (holotype a dried culture GMB 3012); ex-type living culture GMBC 3012.

##### Description.

***Colonies*** on PDA 27–30 mm diam after 14 d at 25 °C, flat, felty at center, creamy white; reverse luteous with a buff margin. ***Mycelium*** consisting of branched, septate, hyaline, smooth- and thin-walled hyphae, 1.5–2.5 μm wide. ***Conidiophores*** aggregated or solitary, erect, straight to curved, arising from aerial or submerged hyphae or from mycelial ropes, unbranched or basitonously branched, bearing 1–3 whorls of 1–3 phialides; sporodochia formed in older cultures; branching pattern verticillate; conidiophores 15–45 × 1–2 μm (x̄ = 28 × 1.5, n = 50), with 1–2 septa, hyaline, smooth-walled, walls thicker than those of vegetative hyphae. ***Phialides*** lateral or terminal, arising laterally from hyphae or formed in small monopodial clusters on conidiophores; subulate, hyaline, thin- and smooth-walled, 14.0–25.5 μm long, 0.9–2.1 μm wide at base (x̄ = 20 × 1.6, n = 50); collarette inconspicuous; polyphialides not observed. ***Conidia*** aseptate, cylindrical, hyaline, thin- and smooth-walled, 3.2–4.8 × 1.5–2.0 μm (x̄ = 3.8 × 1.7, n = 50), aggregated in slimy heads. ***Chlamydospores*** and ***sexual morph*** not observed.

##### Other material examined.

China, • Guizhou Province, Guian New District (same locality and habitat as the holotype), May 2025, Yao Wang (living culture: GMBC 3013).

##### Substrate.

Rhizosphere soil.

##### Distribution.

Known only from Guizhou Province, China.

##### Notes.

*Subuliphorum cylindrosporum* is morphologically similar to *S. camptosporum* (Gams 1971; Hou et al. 2023) but can be distinguished by its shorter phialides (14.0–25.5 × 0.9–2.1 μm, never exceeding 25.5 μm in length, vs. 14.9–69.5 × 0.9–2.2 μm) and larger conidia (3.2–4.8 × 1.5–2.0 μm vs. 2.5–4.0 × 1.1–1.5 μm). In addition, the conidia of *S. cylindrosporum* are consistently cylindrical (Fig. 4C–H), while those of *S. camptosporum* range from short cylindrical to lunate. Phylogenetically, the two species formed a strongly supported sister clade (100%/100%/1; Figs 1, 2) based on combined analyses of concatenated ITS, SSU, LSU, *tef*-*1α*, *rpb1*, and *rpb2* sequences. Pairwise comparisons of sequences from the ex-type strains using BLASTn on NCBI GenBank revealed consistent genetic divergence between the two species for the available loci. As the *rpb1* sequence of *S. camptosporum* is currently unavailable, comparisons focused on ITS, *tef*-*1α*, and *rpb2*. The divergences in ITS (11 bp out of 540 bp, 2.04%) and in the two protein-coding genes (19 bp out of 809 bp, 2.35% in *tef*-*1α*; 26 bp out of 749 bp, 3.47% in *rpb2*) all exceed the >1.5% threshold recommended by Jeewon and Hyde (2016) for species delimitation. The combination of consistent morphological differences and multi-locus genetic divergence exceeding established thresholds strongly supports the recognition of *S. cylindrosporum* as a distinct species.

#### Subuliphorum camptosporum

Taxon classificationFungiHypocrealesClavicipitaceae

(W. Gams) L.W. Hou, L. Cai & Crous, Studies in Mycology 105: 50 (2023)

C1B5C62C-4A48-5DED-970D-6C48863B5C59

845801

Acremonium camptosporum W. Gams, Cephalosporium-artige Schimmelpilze (Stuttgart): 57 (1971) (Basionym).

##### Type.

Germany, • Kiel-Kitzeberg, aerial contaminant, unknown collection date, isol. 1965, coll. and isol. by W. Gams, No. 517, CBS H-6602, CBS H-8108, CBS H-8109, CBS H-8110 & CBS H-8111 (holotype CBS 756.69 preserved as a metabolically inactive culture, ex-type culture CBS 756.69).

##### Descriptions and illustrations.

Hou et al. (2023).

##### Other material examined.

China, • Guizhou Province, Guian New District (26°23'48"N, 106°27'08"E, alt. 1235 m), isolated from the rhizosphere soil of Gaultheria leucocarpa var. yunnanensis, May 2025, Yao Wang (living culture: GMBC 3011).

##### Notes.

Strain GMBC 3011 was identified as *Subuliphorum camptosporum* based on integrated evidence from six-locus phylogenetic analyses and morphological characteristics, both of which are consistent with the taxonomic circumscription of this species by Hou et al. (2023) in all key diagnostic features. This represents both the first report of *S. camptosporum* from a plant rhizosphere habitat and the first geographic record of this species in China, expanding its known ecological amplitude and global distribution. Additionally, we generated the first *rpb1* gene sequence of this species, which fills an important gap in its molecular dataset.

## Discussion

This study advances the taxonomic and ecological understanding of two recently established genera of acremonium-like fungi, *Paraneoaraneomyces* (Zhang et al. 2023) and *Subuliphorum* (Hou et al. 2023). Both genera were originally monotypic, each represented by a single species: *Paraneoaraneomyces sinensis* and *Subuliphorum camptosporum*. Our investigation of the rhizosphere of Gaultheria leucocarpa var. yunnanensis expands their known diversity and ecological range. The discovery and description of the novel species *P. guizhouensis* and *S. cylindrosporum*, together with the first report of *S. camptosporum* from a plant rhizosphere, effectively doubles the species diversity within both genera. This finding also extends the ecological amplitude of *S. camptosporum*, suggesting a broader niche that may include saprotrophic or root-associated lifestyles.

The relatively simple morphology shared by these fungi (hyaline conidial heads produced from solitary or verticillate phialides) makes species delimitation based solely on morphology difficult. A robust molecular phylogenetic framework is therefore essential. Our six-locus analyses (ITS, SSU, LSU, *tef*-*1α*, *rpb1*, and *rpb2*) provide strong resolution of relationships within this lineage. A key phylogenetic finding is the resolution of a sister-group relationship between *Paraneoaraneomyces* and *Subuliphorum*, which received consistently high support across both analytical scales (BS_IQ_/BS_RAx_/PP = 97%/82%/0.99 in the family-wide analysis, 95%/90%/1 in the focused clade analysis; Figs 1, 2), a relationship not previously resolved (Hou et al. 2023; Zhang et al. 2023). Together with *Neoaraneomyces*, these genera form a distinct lineage within Clavicipitaceae, with each genus representing a separate, well-supported clade, thereby reinforcing their generic boundaries (Fig. 1).

Morphological evidence is consistent with this phylogenetic framework. Although these genera share a simplified acremonium-like morphology, they differ in key features of conidiogenesis and conidial morphology (Chen et al. 2022; Hou et al. 2023; Zhang et al. 2023). *Neoaraneomyces* (typified by the spider-pathogenic *N. araneicola*) produces conidia in dry chains, with individual conidia being fusiform to ellipsoidal. In contrast, *Paraneoaraneomyces* species produce cymbiform to reniform conidia on solitary or occasionally whorled phialides, with conidia either adhering at the phialide apex or forming small globose heads. *Subuliphorum* is distinguished by verticillately branched conidiophores and by cylindrical to lunate conidia that aggregate into slimy heads. These consistent differences in conidial arrangement (chains vs. distinct heads) and conidial shape provide robust morphological support for their phylogenetic distinctness and the recognition of three separate genera within this lineage (Chen et al. 2022; Hou et al. 2023; Zhang et al. 2023).

The morphological differences among these genera appear to correlate with their distinct ecological niches. The dry, chain-borne conidia of *Neoaraneomyces* facilitate aerial dispersal, a feature consistent with its obligate spider-pathogenic lifestyle that requires locating mobile arthropod hosts (Chen et al. 2022). In contrast, the non-slimy globose conidial heads of *Paraneoaraneomyces* and the slimy conidial heads of *Subuliphorum* both enhance adhesion to soil particles and plant root surfaces (Hou et al. 2023; Zhang et al. 2023), which may represent convergent adaptations for the colonization of rhizosphere soil habitats. The recovery of all three taxa from the same rhizosphere microhabitat suggests that such environments may selectively favor clavicipitaceous fungi with root-adherent conidial traits. That two novel species and a new habitat record were obtained from a single plant species and sampling site further underscores that targeted sampling of underexplored microhabitats represents an effective strategy for uncovering hidden fungal diversity.

Several limitations of this study should be acknowledged. First, the sampling was restricted to a single geographic location in Guizhou Province, a single plant species, and a single time point, which limits the generalizability of our findings, particularly regarding the ecological distribution of these rare fungi. Second, no functional experiments were conducted to clarify the specific interactions between these fungi and Gaultheria leucocarpa var. yunnanensis. Additionally, the lack of *rpb1* gene sequences for some closely related species may affect the resolution of our phylogenetic analyses to some extent. Future studies incorporating broader geographic and plant species sampling across multiple seasons, combined with metagenomic profiling and culture-based functional assays, will be essential to fully elucidate the ecological roles and evolutionary history of these understudied clavicipitaceous lineages.

In summary, this study expands the diversity and ecological understanding of *Paraneoaraneomyces* and *Subuliphorum*, clarifies their phylogenetic relationships, and reinforces their taxonomic boundaries within Clavicipitaceae. The discovery of these fungi in the rhizosphere underscores the value of exploring underexplored microhabitats in revealing hidden fungal diversity. Continued investigation of such niches will be essential for refining the systematics and ecological understanding of acremonium-like fungi and other microfungal groups.

## Supplementary Material

XML Treatment for Paraneoaraneomyces guizhouensis

XML Treatment for Subuliphorum cylindrosporum

XML Treatment for Subuliphorum camptosporum

## Additional information

### Artificial Intelligence (AI) use

The authors accept full responsibility for the content of the manuscript, including the disclosure of any use of AI.

No AI tools were used in the preparation of this manuscript.

### Author ORCIDs

Y. Wang https://orcid.org/0000-0002-1262-6700

H. Chen https://orcid.org/0009-0008-0291-3571

### Data availability

All of the data that support the findings of this study are available in the main text or Supplementary Information.

## Supplementary materials

Supplementary material 1 Supplementary figures Data type doc Explanation note **fig. S1**. Maximum Likelihood phylogenetic tree inferred from nr*SSU* sequences, showing the relationships among all available strains of *Paraneoaraneomyces* and *Subuliphorum*. **fig. S2**. Maximum Likelihood phylogenetic tree inferred from ITS sequences, showing the relationships among all available strains of *Paraneoaraneomyces*, *Subuliphorum*, and *Neoaraneomyces*. **fig. S3**. Maximum Likelihood phylogenetic tree inferred from nr*LSU* sequences, showing the relationships among all available strains of *Paraneoaraneomyces*, *Subuliphorum*, and *Neoaraneomyces*. **fig. S4**. Maximum Likelihood phylogenetic tree inferred from *tef*-*1α* sequences, showing the relationships among all available strains of *Paraneoaraneomyces*, *Subuliphorum*, and *Neoaraneomyces*. **fig. S5**. Maximum Likelihood phylogenetic tree inferred from *rpb1* sequences, showing the relationships among all available strains of *Paraneoaraneomyces*, *Subuliphorum*, and *Neoaraneomyces*. **fig. S6**. Maximum Likelihood phylogenetic tree inferred from *rpb2* sequences, showing the relationships among all available strains of *Paraneoaraneomyces*, *Subuliphorum*, and *Neoaraneomyces*. https://binary.pensoft.net/file/1670743This dataset is made available under the Open Database License (http://opendatacommons.org/licenses/odbl/1.0/). The Open Database License (ODbL) is a license agreement intended to allow users to freely share, modify, and use this Dataset while maintaining this same freedom for others, provided that the original source and author(s) are credited. Yao Wang, Zhi-Qin Wang, Hui Chen, Li-Ping Zhu, Fan Yang, Shao-Huan Liu
