# Supplementary material for: Discovery of three rare acremonium-like fungi in the rhizosphere of Gaultheria leucocarpa var. yunnanensis resolves the sister relationship between Paraneoaraneomyces and Subuliphorum (Clavicipitaceae, Hypocreales)
Source: MycoKeys. 2026 Jun 5;133:277–300. doi: 10.3897/mycokeys.133.192083 (PMC13263725; doi:10.3897/mycokeys.133.192083)
Supplement: Supplementary material 1 — Supplementary figures [file mycokeys-133-277-s001.doc]

**

**

**Figure S1.** Maximum Likelihood phylogenetic tree inferred from nr*SSU* sequences, showing the relationships among all available strains of *Paraneoaraneomyces* and *Subuliphorum*. Bootstrap support values (BS) ≥75% are shown at nodes. Strains examined in this study are highlighted in bold. Note that *Neoaraneomyces* sequences were not available for this locus.

**

**

**Figure S2.** Maximum Likelihood phylogenetic tree inferred from ITS sequences, showing the relationships among all available strains of Paraneoaraneomyces, Subuliphorum, and Neoaraneomyces. Bootstrap support values (BS) ≥75% are shown at nodes. Strains examined in this study are highlighted in bold.





**Figure S3.** Maximum Likelihood phylogenetic tree inferred from nr*LSU* sequences, showing the relationships among all available strains of Paraneoaraneomyces, Subuliphorum, and Neoaraneomyces. Bootstrap support values (BS) ≥75% are shown at nodes. Strains examined in this study are highlighted in bold.





**Figure S4.** Maximum Likelihood phylogenetic tree inferred from *tef-1α* sequences, showing the relationships among all available strains of Paraneoaraneomyces, Subuliphorum, and Neoaraneomyces. Bootstrap support values (BS) ≥75% are shown at nodes. Strains examined in this study are highlighted in bold.

*

*

**Figure S5.** Maximum Likelihood phylogenetic tree inferred from *rpb1* sequences, showing the relationships among all available strains of Paraneoaraneomyces, Subuliphorum, and Neoaraneomyces. Bootstrap support values (BS) ≥75% are shown at nodes. Strains examined in this study are highlighted in bold.





**Figure S6.** Maximum Likelihood phylogenetic tree inferred from *rpb2* sequences, showing the relationships among all available strains of Paraneoaraneomyces, Subuliphorum, and Neoaraneomyces. Bootstrap support values (BS) ≥75% are shown at nodes. Strains examined in this study are highlighted in bold.
